# Effects of Rapid Thermal Annealing and Different Oxidants on the Properties of La_x_Al_y_O Nanolaminate Films Deposited by Atomic Layer Deposition

**DOI:** 10.1186/s11671-017-1994-z

**Published:** 2017-03-23

**Authors:** Chenxi Fei, Hongxia Liu, Xing Wang, Lu Zhao, Dongdong Zhao, Xingyao Feng

**Affiliations:** Key Laboratory for Wide-Band Gap Semiconductor Materials and Devices of Education, School of Microelectronics, Xidian University, Xi’an, 710071 China

**Keywords:** ALD, X-ray photoelectron spectroscopy, Rapid thermal annealing, EOT

## Abstract

A comparative study of different sequences of two metal precursors [trimethylaluminum (TMA) and Tris(isopropylcyclopentadienyl) lanthanum (La(^i^PrCp)_3_)] for atomic layer deposition (ALD) lanthanum aluminum oxide (La_x_Al_y_O) films is carried out. The percentage compositions of C and N impurity of La_x_Al_y_O films were investigated using in situ X-ray photoelectron spectroscopy (XPS). The effects of different oxidants on the physical and chemical properties and electrical characteristics of La_x_Al_y_O films are studied before and after annealing. Preliminary testing results indicate that the impurity level of La_x_Al_y_O films grown with different oxidants can be well controlled before and after annealing. Analysis indicates the rapid thermal annealing (RTA) and kinds of oxidants have significant effects on the equivalent oxide thickness (EOT), dielectric constant, electrical properties, and stability of La_x_Al_y_O films. Additionally, the change of chemical bond types of rapid thermal annealing effects on the properties of La_x_Al_y_O films are grown with different oxidants also investigated by XPS.

## Background

The miniaturization of complementary metal-oxide-semiconductor (CMOS) devices would eventually require the thinning of a gate dielectric, whose capacitance should be equivalent to that of SiO_2_ with a thickness less than 1 nm. Ultrathin SiO_2_ as a gate dielectric has been rapidly confronted with its fundamental limit due to its unacceptably high leakage current. The replacement of SiO_2_ with high dielectric constant (*k*) materials has recently attracted considerable attention because their large physical thickness can suppress a gate tunneling leakage current at a scaled equivalent oxide thickness (EOT) [1–4]. Several candidate materials for the gate dielectric films such as HfO_2_ [5], ZrO_2_ [6], La_2_O_3_ [7], Y_2_O_3_ [8], Ta_2_O_5_ [9], and Al_2_O_3_ [10] have been studied extensively during the past decade. As a promising high-*k* material, La_2_O_3_ has advantages of high dielectric constant (~30) and good thermal stability, but the hygroscopicity would lead to high leakage [11]. Al_2_O_3_ has many favorable properties like kinetic stability and thermodynamic stability on Si up to high temperatures, good interface with Si, and low bulk defect density. However, the dielectric constant of Al_2_O_3_ (~9) is low [12]. Lanthanum aluminate (LaAlO_3_ or LAO), which is a compound of La_2_O_3_ and Al_2_O_3_, is a promising material as it possesses a large bandgap (5–6 eV), a high dielectric constant (22–25), and a relatively large band offset with Si (2 eV)[13].

Various deposition techniques such as molecular beam epitaxy (MBE) [14], pulsed laser deposition (PLD) [15], metal-organic chemical vapor deposition (MOCVD) [16], and atomic layer deposition (ALD) have been explored to grow the high-*k* dielectric layers on Si substrate [17]. ALD is a method of cyclic deposition and oxidation which consists of alternate pulsing of the precursor gases and vapors on the substrate surface resulting in subsequent chemisorptions or surface reaction of the precursors to produce the desired oxide. Under suitable conditions, ALD is a saturation reaction with constant thickness increase in each deposition cycle. Hence, regardless of the precursor dose supplied, the resulting thickness will always be the same, if the appropriate saturation dose is supplied. This is termed as the self-limiting growth mechanism of ALD which facilitates the growth of conformal thin films with accurate thickness control. ALD is also suitable for depositions on trench-type structures. Also, for thin films, the ALD produces better uniformity and lesser defects as compared to other deposition techniques [18, 19]. These qualities make the ALD method attractive for manufacturing of future generation integrated circuits especially gate dielectric applications.

Various oxygen sources have been used in the past for ALD such as O_3_, O_2_, and, the most common, H_2_O. The oxidation power of the oxygen source towards the bare Si surface is very important in ALD to achieve low EOT values, since growth of low *k* layer such as SiO_x_ can reduce the overall capacitance. In order to obtain low charge leakage, residual impurities in the high-*k* film should be reduced as much as possible. The oxidants have great influences on the defects and residual impurities in the high-*k* film if the process conditions are optimized in ALD process. On the other hand, the effects of RTA on the properties of La_x_Al_y_O films have been reported [20, 21]. People found that the growth of the interface layer was suppressed and the properties of film were enhanced by RTA. However, the oxidant effects on the characteristics of La_x_Al_y_O before and after annealing have rarely been discussed. In this study, two kinds of oxidants (H_2_O and O_3_) are used to deposit La_x_Al_y_O films by ALD. The effects of the different combinations of the different oxidants with metal precursors on the physical, electrical, and chemical properties of annealed La_x_Al_y_O films are investigated.

## Methods

**Fig. 1:**
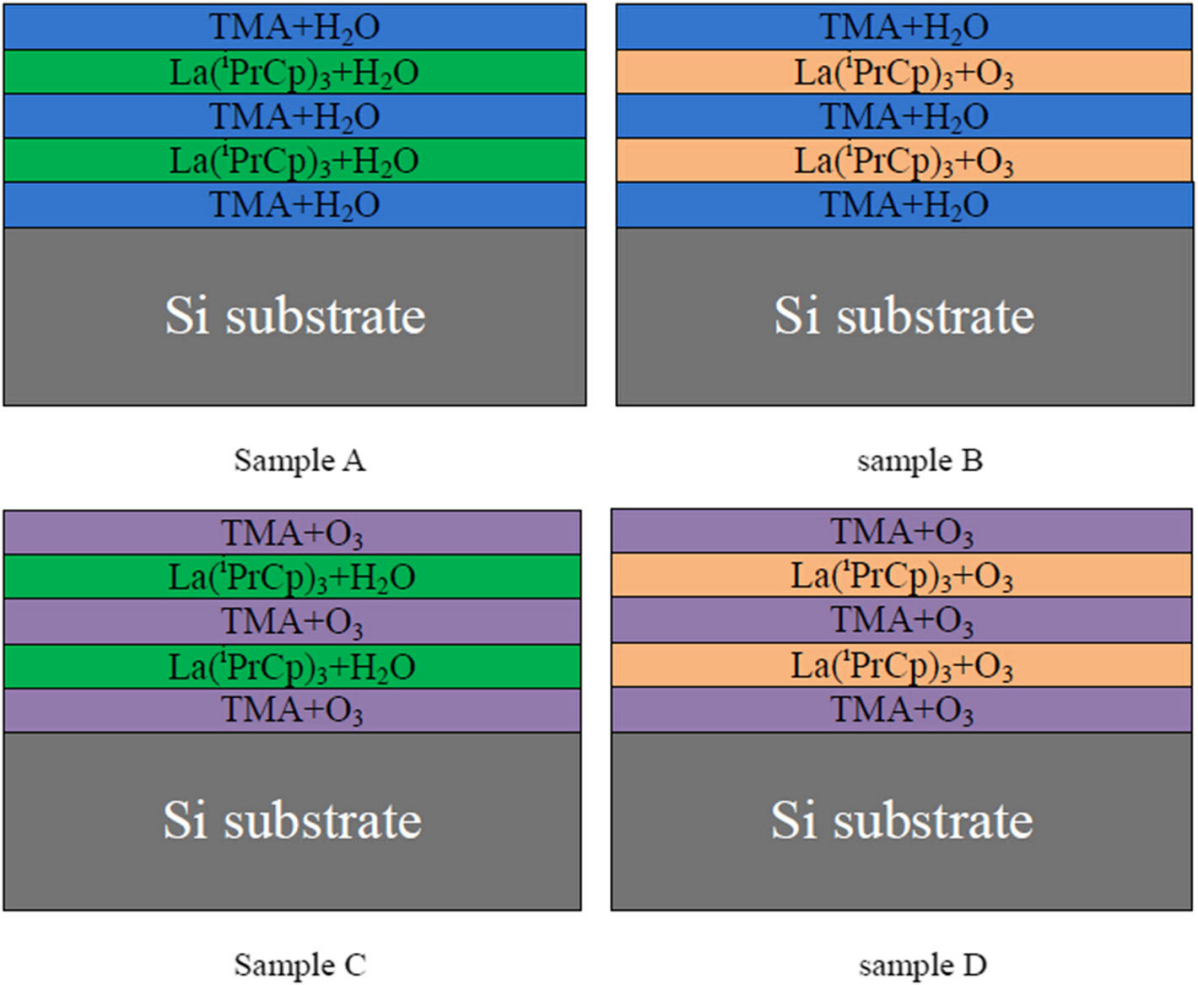
Schematic drawings of structures of different La_x_Al_y_O films

A P-type Si B-doped (100) wafer with a resistivity of 8–12 Ω cm was cleaned with a (HCl:H_2_O_2_:H_2_O = 1:1:5) solution for 10 min at 85 °C to remove organic contamination and then chemically etched with a diluted hydrofluoric acid solution (HF:H_2_O = 1:100) to remove native oxides, both followed by a 30-s rinse in deionized water. La_x_Al_y_O films were deposited on Si wafers by a layer-by-layer deposition process using different metal processors (trimethylaluminum (TMA) and tris(isopropylcyclopentadienyl) lanthanum [La(^i^PrCp)_3_]) and oxidants (H_2_O and O_3_) at 300 °C by ALD reactor (Picosun R-200, Espoo, Finland). Ultra-high purity nitrogen (N_2_, 99.999%) was employed as carrier and purge gas. The container of the TMA is at room temperature, corresponding to a vapor pressure of 10 to 15 hPa, and La(^i^PrCp)_3_ was kept at 180 °C, respectively. When H_2_O is used as an oxidant, ALD sequence was composed of 0.5 s La(^i^PrCp)_3_ pulse/6 s purge with N_2_/0.5 s H_2_O pulse/8 s purge with N_2_ and 0.1 s TMA pulse/3 s purge with N_2_/0.1 s H_2_O pulse/4 s purge with N_2_. When O_3_ is used as an oxidant, ALD sequence was composed of 0.5 s La(^i^PrCp)_3_ pulse/8 s purge with N_2_/0.5 s O_3_ pulse/10s purge with N_2_ and 0.1 s TMA pulse/3 s purge with N_2_/0.5 s O_3_ pulse/4 s purge with N_2_. Post-deposition annealing (PDA) was performed for 60 s in N_2_ ambient at 600 °C using rapid thermal annealing (RTA). Figure 1 shows the schematic drawings of structures of different La_x_Al_y_O films. The number of deposition cycles for all films were 100. Film thicknesses were measured by J.A. Woollam M2000D spectroscopic ellipsometry. The bonding structures of the films were examined by X-ray photoelectron spectroscopy (XPS). The electrical properties of the films were measured using a metal-insulator semiconductor (MIS) capacitor structure. Metal gate (200 nm Au) with a diameter of 130 μm was deposited by magnetron sputtering through a shadow mask, and Al was sputtered as the back contact metal. Capacitance-voltage (*C*-*V*) characteristics were evaluated using an Agilent B1500A semiconductor parameter analyzer. The EOT and dielectric constant of the capacitor were obtained by the Hauser CVC program taking into account quantum mechanical effects.

## Results and Discussion

**Fig. 2:**
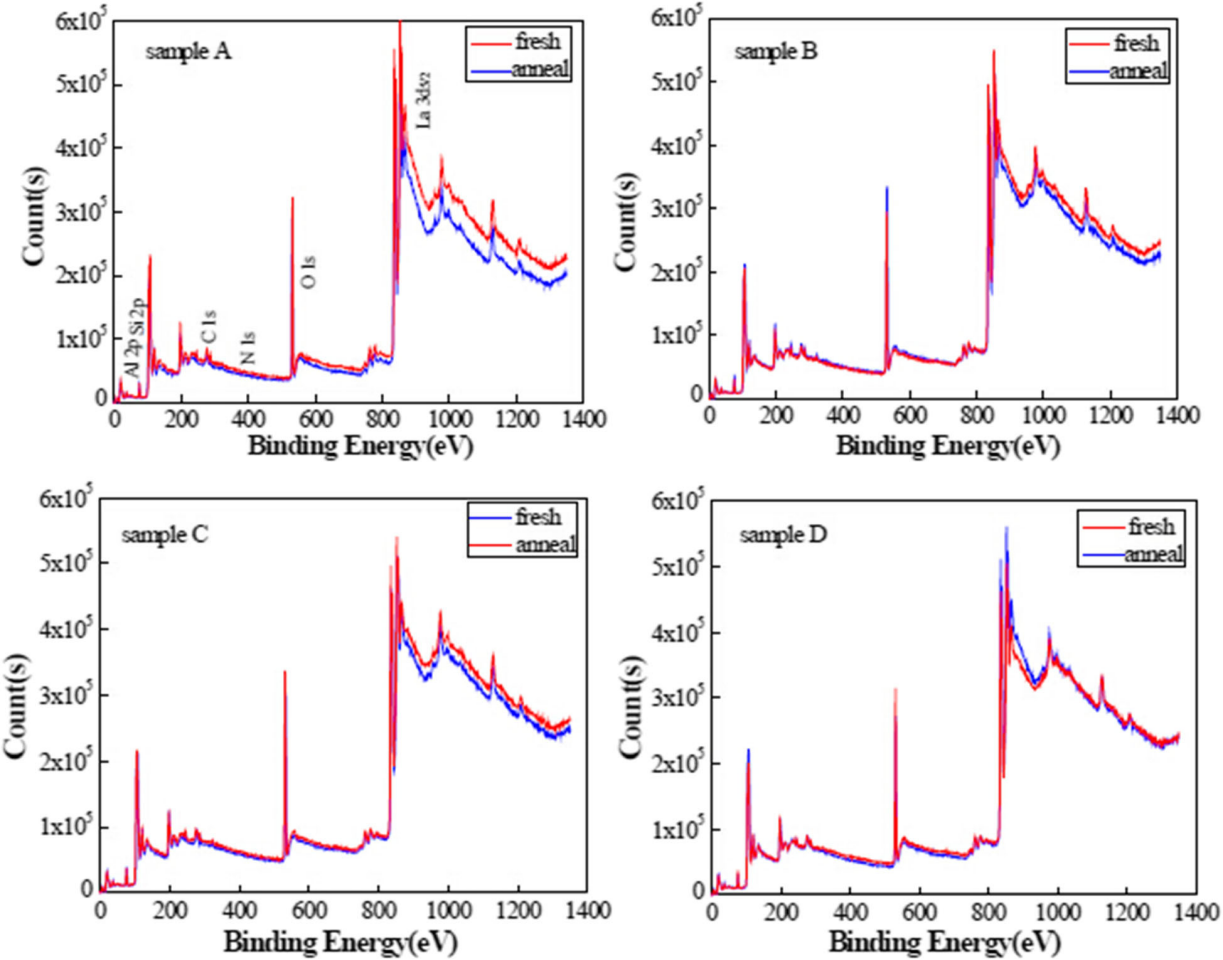
XPS spectra of La_x_Al_y_O films grown using different oxidants before and after annealing

Figure 2 shows the XPS spectra of the La_x_Al_y_O films with different oxidants before and after annealing. The main peaks consist of Al 2p, O 1 s, and La 3d_5/2_ peaks; subordinate peaks consist of C 1 s, N 1 s, and Si 2p peaks. The interactions between La_2_O_3_ and Al_2_O_3_ layers occur, which is accompanied with the decomposition and recomposition of unstable bonds or groups residing in La_x_Al_y_O films during the annealing process. Therefore, the change is observed in the XPS spectrum of the La_x_Al_y_O films after annealing. On the other hand, an obvious change is observed in the spectrum of sample A after annealing compared to the other samples. This phenomenon attributed to the physical adsorption property of H_2_O. The high-concentration hydroxyl/hydrogen groups were formed in La_x_Al_y_O films although the purge time is sufficiently long during the ALD process. The residue of hydroxyl/hydrogen groups generated many defects and dangling bonds, which causes the increasing of the impurity residuals in La_x_Al_y_O films. In contrast to the H_2_O, O_3_ preserves the self-limiting nature of ALD reactions, and no oxidant by-products reside in film growth. Therefore, the change is not obvious in the spectrum of sample D after annealing.

**Fig. 3:**
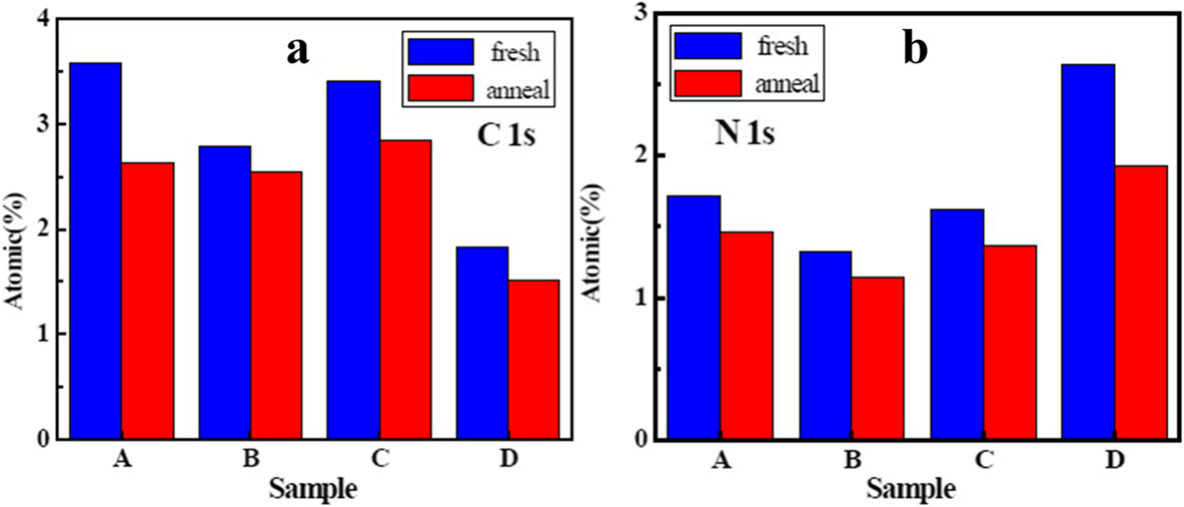
Percentage compositions of atoms in La_x_Al_y_O films before and after annealing. **a** C. **b** N

The XPS quantitative analysis is performed to determine the chemical composition of the film. Figure 3 shows the percentage compositions of C and N impurities for La_x_Al_y_O films. The residual C impurity mainly comes from the residues of metal precursors or C-containing groups attached to the metal atoms for the as-deposited samples. In Fig. 3a, the percentage composition of C impurity for as-deposited sample A is higher than that for the other samples. Moreover, the variation of the degree of reduction of the percentage composition of C impurity for sample A is more severe than that for the other samples after annealing. This result indicates that the film using O_3_ as an oxidant is more prone to achieve the saturation adsorption reaction and has a greater advantage in controlling the C impurity level compared with the film using H_2_O as an oxidant [19]. On the other hand, the percentage composition of N impurity for as-deposited sample D is higher than that for the other samples shown in Fig. 3b. The residual N impurity mainly comes from the formation of La–N and Si–N bonding. O_3_ with strong oxidization and lability can split the N–C bonds of by-products and ligands easily. The unsaturated dangling bonds attach to La in the deposition process and form La–N bonds, which is defined as residual N-related impurities. Due to the diffusivity of the atoms, furthermore, Si–N bonds are formed at the interface between the film and Si substrate in the deposition process. The two reasons explain the phenomenon that the percentage composition of N impurity of sample D is higher than that of the other samples. During the annealing process, the unstable bonds can decompose, and carrier gas purges the residue out of the chamber which caused the decreasing of the content of N impurity [22].

Table 1 shows the percentage compositions of different atoms in different La_x_Al_y_O films. The ratio of La:Al:O approximately 1:3:6 for each samples before and after annealing indicates that the oxidants have a small influence on the stoichiometry of La_x_Al_y_O films. In conclusion, the La_x_Al_y_O film grown with O_3_ as the oxidant generates lower C and higher N impurity level than the films using H_2_O as an oxidant. C and N impurity concentrations decreased, and the characteristics of La_x_Al_y_O films improved after rapid thermal annealing.

**Table 1:** Percentage compositions of atomic atoms in La_x_Al_y_O films

Sample		La(at%)	Al(at%)	O(at%)	C(at%)	N(at%)
Sample A	Fresh	11.99	26.37	56.35	3.58	1.71
	Anneal	11.28	27.14	57.48	2.64	1.46
Sample B	Fresh	11.96	26.69	57.24	2.79	1.32
	Anneal	9.98	27.18	59.15	2.55	1.14
Sample C	Fresh	11.24	26.04	57.69	3.41	1.62
	Anneal	11.03	26.58	58.17	2.85	1.37
Sample D	Fresh	9.75	27.17	58.61	1.83	2.64
	Anneal	8.91	27.78	59.87	1.51	1.93

**Fig. 4:**
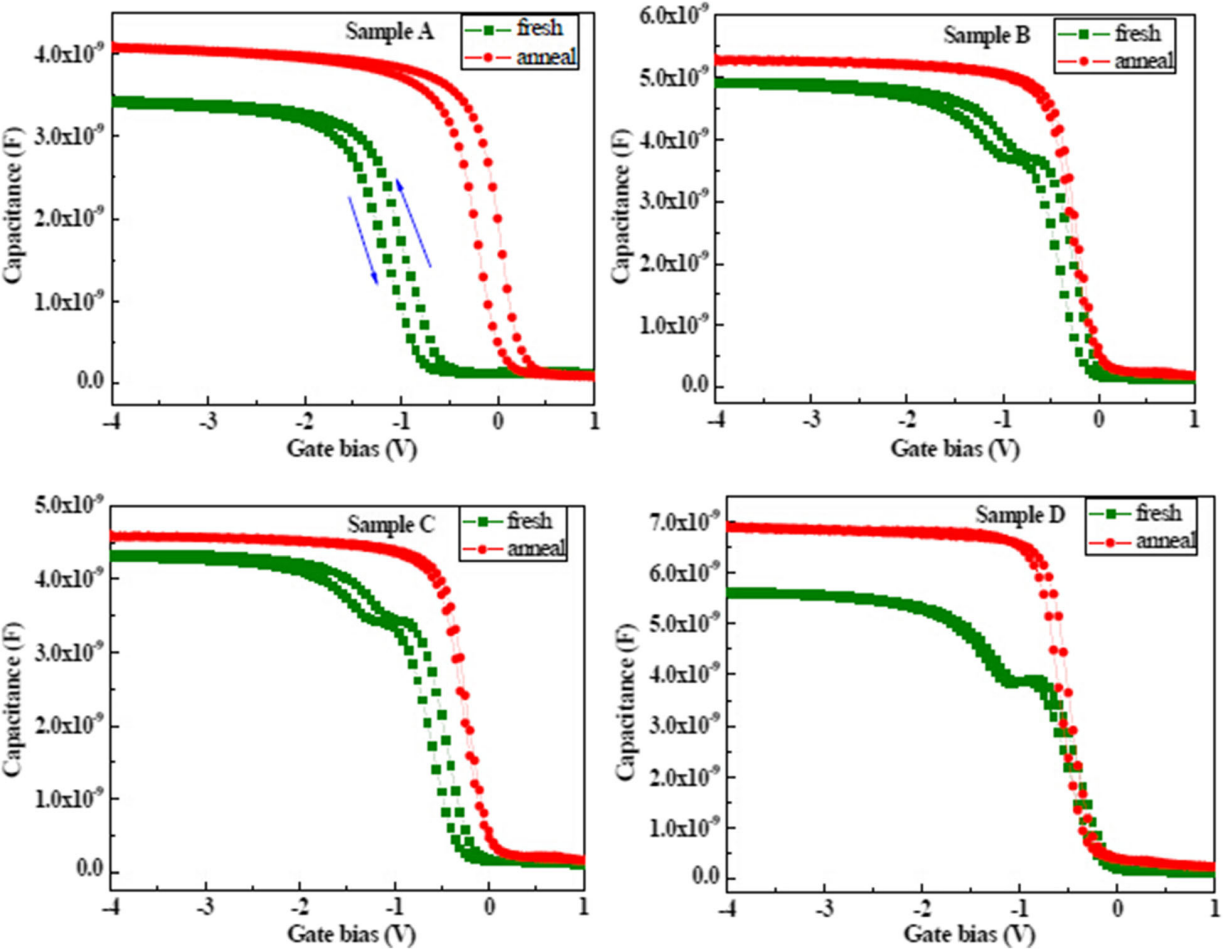
The *C*-*V* characteristics of La_x_Al_y_O films before and after annealing

Figure 4 shows *C*-*V* characteristics of the La_x_Al_y_O films with different oxidants before and after annealing. The gate voltage was swept from negative to positive voltage and then again back to negative voltage. *C*-*V* curves with O_3_ used as oxidant formed a width step which is caused by the trapped holes injected from the La_x_Al_y_O layer into the depletion layer. The width of the depletion layer in the Si substrate grows with the gate bias increasing. Growth of the depletion layer will stop, and the capacitance becomes constant after all the trapped holes in the interface layer are injected into the depletion layer. Magnitude of the trap charge concentration in the oxide layer is determined by the magnitude of the hysteresis voltage. Sample D has a small hysteresis voltage compared with the other samples before annealing. This indicates that film using O_3_ as an oxidant possesses low-interface state density and good-interface quality. For samples A, B, and C, oxidant contains water, and the complex interface layer (IL) is formatted due to the interdiffusion of Si, Al, La, and O atoms in the deposition process. Moreover, the values of flat band voltage (V_FB_) of four samples are negative before annealing. This attributed to the formation of positive fixed charges in films. The formation of oxygen vacancies in La_x_Al_y_O films increases the positive oxide charge due to the growth of silicate interfacial layer. The flat band voltage was shifted in a positive direction, and the hysteresis voltages decreased after annealing at 600 °C for La_x_Al_y_O films. The quantity of positive charges is reduced, and oxygen vacancies are filled due to the decomposition and recombination of chemical bonds in films during the annealing process [23]. This indicates that La_x_Al_y_O films possess a lower trap charge density and a better quality after annealing.

On the other hand, the values of accumulation capacitance of films increased after annealing; this attributed to a decrease of the concentration of interfacial fixed charge and defects. However, sample D has a large value of accumulation capacitance compared with the other samples before and after annealing. There are two reasons for this phenomenon. First of all, films deposited with oxidant containing H_2_O tend to easily form La(OH)_3_ which will decrease the whole dielectric constant and value of accumulation capacitance of films. Secondly, the use of O_3_ as the oxidant improved electrical properties of the La_x_Al_y_O films by suppressing the formation of complex interfacial layers and La(OH)_3_[24].

**Fig. 5:**
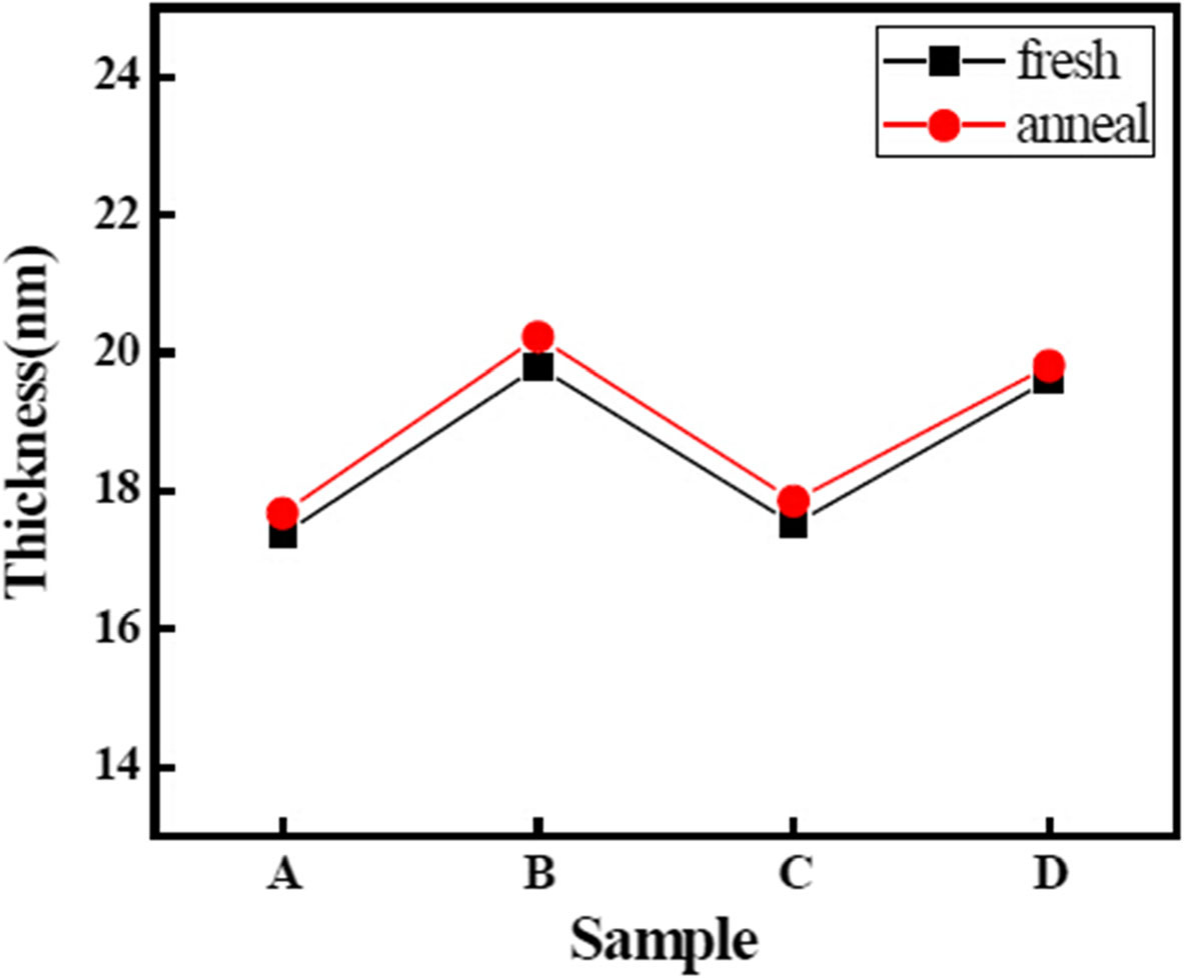
Values of thickness of the La_x_Al_y_O films before and after annealing

Figure 5 shows the values of thickness of the La_x_Al_y_O films. The values of thickness of samples B and D are higher than the values of thickness of samples A and C. This indicates that the growth rates of La_2_O_3_ and Al_2_O_3_ films using O_3_ as an oxidant are higher than the films using H_2_O as an oxidant. Growth rates of Al_2_O_3_ films achieved stable values of 0.97 and 1.01 Å/cycle when H_2_O is used as oxidant, and growth rates of La_2_O_3_ films achieved stable values of 0.71 and 1.03 Å/cycle when O_3_ is used as oxidant. This result indicates that the film using O_3_ as an oxidant is more prone to achieve the saturation adsorption reaction. This analysis is in accord with the analyses of changes of impurity content we discussed before. Furthermore, the values of thickness of the La_x_Al_y_O films increased after annealing because of the formation of an interfacial layer between the film growth layer and Si substrate. Moreover, the change of values of thickness of four La_x_Al_y_O films before and after annealing is negligible. This can be explained by the densification of the films after thermal annealing [25].

**Fig. 6:**
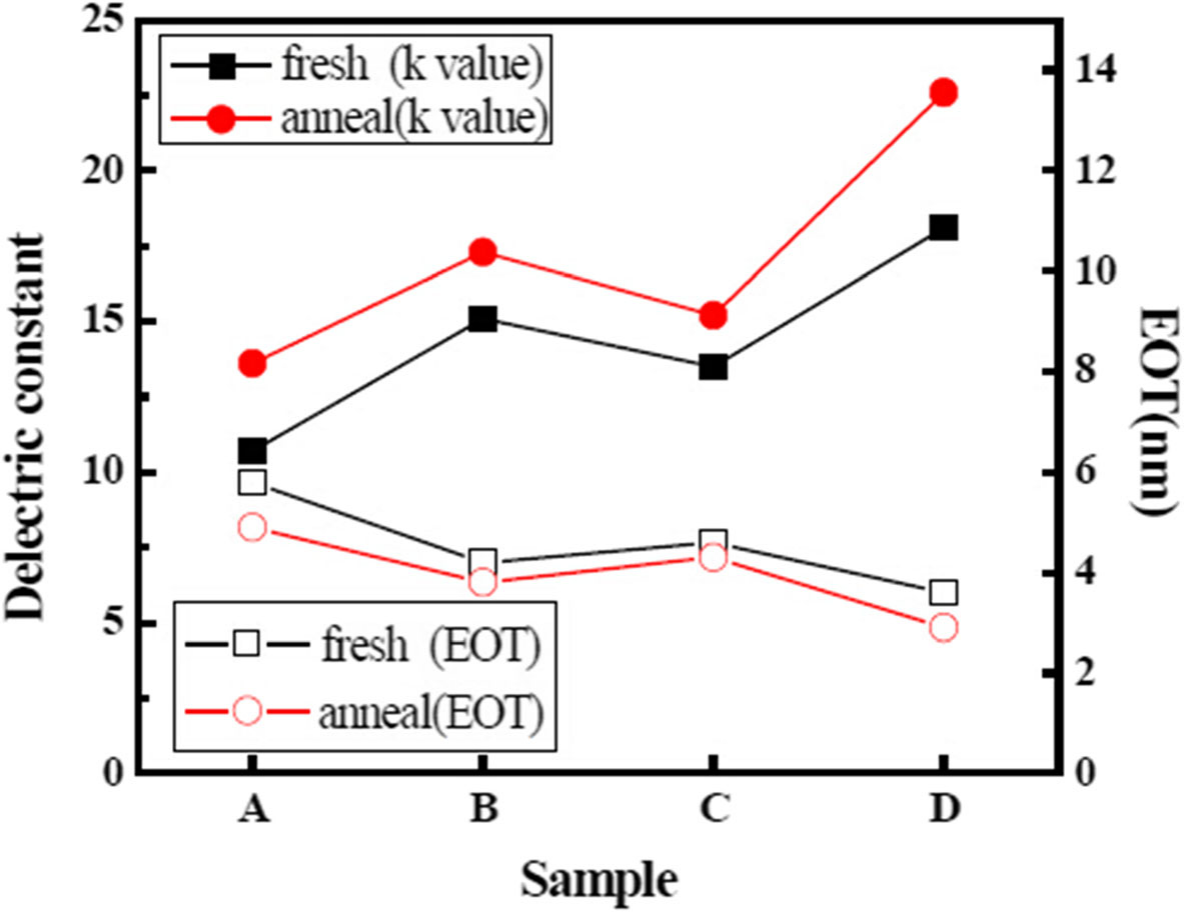
Values of dielectric constant and EOT of the La_x_Al_y_O films before and after annealing

Figure 6 shows the values of dielectric constant and EOT of the La_x_Al_y_O films. As shown in Fig. 6, the *k* value and EOT of sample A are 10.7 and 5.8 nm, respectively. Sample A has a small permittivity and large EOT compared with the other samples. The small permittivity attributed to the formation of La(OH)_3_ and La silicate during the ALD process. Formation of much La(OH)_3_ is because of the reaction of La_2_O_3_ layer and the moisture contained in H_2_O oxidant, carrier gas, and atmosphere [26]. Formation of La silicate attributed to the interdiffusion of La and O atoms that belong to La_2_O_3_ layer close to substrate and Si atoms which came from Si substrate. The *k* value and EOT are 18.1 and 3.6 nm of sample D, which has the largest permittivity and smallest EOT. This phenomenon attributed to the advantage of O_3_. Different from H_2_O, the use of O_3_ as an oxidant can suppress the formation of La(OH)_3_. This caused sample D possessing large permittivity. Values of EOT and *k* value of sample B are 4.2 nm and 15.1, respectively; values of EOT and *k* value of sample C are 4.6 nm and 13.5, respectively. For the two samples, both with mixed as oxidants, possess different properties. For sample C, the La precursor reacts with O_3_ while the Al precursor reacts with H_2_O in the deposition process. For sample B, on the contrary, the La precursor reacts with H_2_O, while the Al precursor reacts with O_3_. The different deposition sequences of oxidants for H_2_O and O_3_ cause sample C formatting less La(OH)_3_ and better interface layer quality which attributed to the reaction of saturated adsorption in La_2_O_3_ films and less diffusion between atoms at interface compared with B. The phenomenon makes sample C possessing larger permittivity and smaller EOT than sample B.

EOT decreased and permittivity increased of the four samples after annealing at 600 °C. The reduction of EOT mainly attributed to the densification process of La_x_Al_y_O films. The La, Si, and O atoms recombined in interface layer during the RTA process; this caused a decrease of the concentration of interfacial fixed charge and defects [27]. Furthermore, the increasing of permittivity attributed to the increasing of accumulation capacitance and reduction of impurity after annealing.

**Fig. 7:**
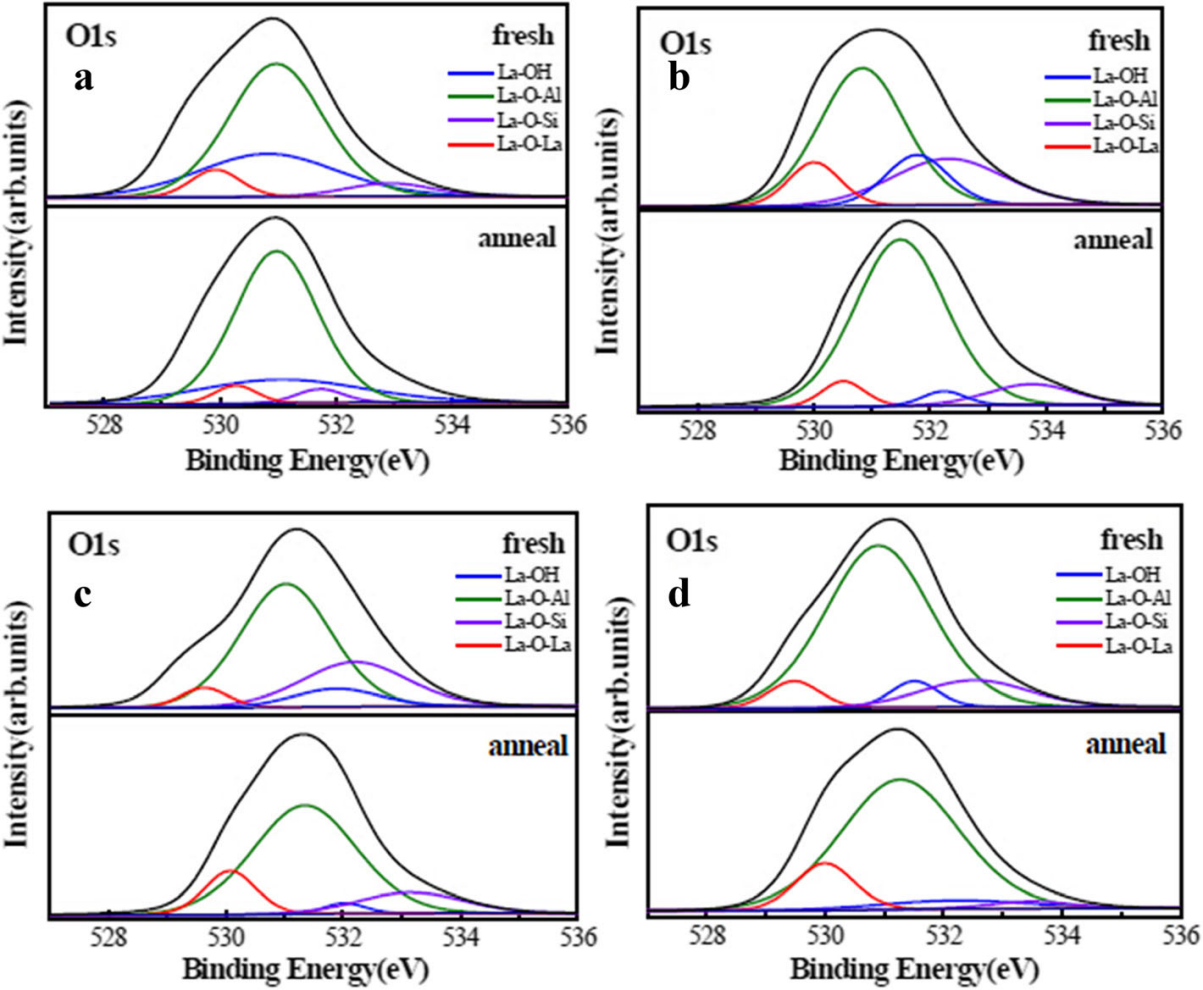
O 1 s XPS peaks before (As Dep) and after annealing for samples. **a** Sample A. **b** Sample B. **c** Sample C. **d** sample D

In order to prove the analyses above, XPS spectra were obtained using Al Kα. The binding energy (BE) was calibrated with the position of the C 1 s peak at 284.8 eV. O 1 s spectrums of four samples before and after annealing were fitted with four peaks after the application of a Smart background are shown in Figure 7. Red, green, blue, and purple curves stand for the La–O–La, La–O–Al, La–OH, and La–O–Si bonds, respectively. The existence of La–O–Al and La–O–La bonds attributed to the formation of La_x_Al_y_O and La_2_O_3_ layer in films. The existence of La–O–Si bond indicates the formation of La silicate at La_2_O_3_/Si interfacial layer [28]. According to previous report, La atom has the strongest tendency among rare earth atoms forming silicate components [29]. Thus, the first few cycles of ALD La_2_O_3_ are consumed to form a silicate interlayer. As shown in Fig. 7a, sample A possesses a large intensity of La–OH peak which attributed to the La(OH)_3_ compared with the other samples. This phenomenon indicates that the film with H_2_O used as oxidant more easily leads to the formation of La hydroxide and reduction of permittivity. As shown in Fig. 7b, c, the intensities of La–OH and La–O–Si peaks of sample B are larger than that of sample C. This difference indicates that sample B has a large EOT and a small permittivity compared with sample C, which coincides with the values of EOT and permittivity for corresponding samples.

Moreover, the intensities of La–OH and La–O–Si peaks of La_x_Al_y_O films decreased after annealing at 600 °C. This attributed to the reduction of impurity content and concentration of defects in the interface of films during the annealing process. Sample D has a smallest intensity of La–OH and La–O–Si peaks compared with the other samples after annealing shown in Fig. 7d. This indicates that the use of O_3_ as the oxidant suppressed the formation of La(OH)_3_ and growth of interface layer. To summarize, annealing improved the electrical properties and increased the permittivity of La_x_Al_y_O films.

## Conclusions

In summary, the annealing effect of La_x_Al_y_O nanolaminate films with different oxidants (H_2_O and O_3_) deposited on a Si substrate by ALD was investigated. First of all, the C and N impurity concentrations in La_x_Al_y_O films were improved by rapid thermal annealing. Moreover, electrical properties were improved of films, and content of La hydroxide was reduced by rapid thermal annealing, which makes films to have a large dielectric constant and a small EOT. Furthermore, the use of H_2_O as the oxidant leads to the formation of La(OH)_3_, which makes the properties of films worse. Using O_3_ as the oxidant improved electrical properties of the deposited La_x_Al_y_O films by suppressing the formation of interface layer and La(OH)_3_. The La_x_Al_y_O film using O_3_ as an oxidant possessed a high permittivity and a small EOT compared with the other samples after annealing. These results show that using O_3_ as an oxidant is suitable for high-performance ALD La_x_Al_y_O film deposition as required for gate dielectric applications.

## Abbreviations

ALD: Atomic layer deposition
CMOS: Complementary metal-oxide-semiconductor
EOT: Equivalent oxide thickness
IL: Interfacial layer
La(^i^PrCp)_3_: Tris(isopropylcyclopentadienyl) lanthanum
MBE: Molecular beam epitaxy
MIS: Metal-insulator semiconductor
MOCVD: Metal-organic chemical vapor deposition
PDA: Post-deposition annealing
PLD: Pulsed laser deposition
RTA: Rapid thermal annealing
TMA: Trimethylaluminum
XPS: X-ray photoelectron spectroscopy

## References

[1] Gray NW, Prestgard MC, Tiwari A (2014). Tb_2_O_3_ thin films: an alternative candidate for high-k dielectric applications. Appl Phys Lett.

[2] Wong H, Yang BL, Dong S, Iwai H, Kakushima K, Ahmet P (2012). Current conduction and stability of CeO_2_/La_2_O_3_ stacked gate dielectric. Appl Phys Lett.

[3] Terlinden NM, Dingemans G, Vandalon V, Bosch RHEC, Kessels WMM (2014). Influence of the SiO_2_ interlayer thickness on the density and polarity of charges in Si/SiO_2_/Al_2_O_3_ stacks as studied by optical second-harmonic generation. J Appl Phys.

[4] Cao D, Cheng X, Jia T, Xu D, Wang Z, Xia C (2013). Characterization of HfO_2_/ La_2_O_3_ layered stacking deposited on si substrate. J Vac Sci Technol B.

[5] Sharma A, Longo V, Verheijen MA, Bol AA, (Erwin) Kessels WMM. Atomic layer deposition of HfO_2_ using HfCp(NMe_2_)_3_ and O_2_ plasma. J. Vac. Sci. Technol. A. 2017; 35:01B130

[6] Weinreich W, Wilde L, Müller J, Sundqvist J, Erben E, Heitmann J (2013). Structural properties of as deposited and annealed ZrO_2_ influenced by atomic layer deposition, substrate, and doping. J Vac Sci Technol A.

[7] Wang X, Liu HX, Fei CX, Yin SY, Fan XJ (2015). Silicon diffusion control in atomic-layer-deposited Al_2_O_3_/La_2_O_3_/Al_2_O_3_ gate stacks using an Al_2_O_3_ barrier layer. Nanoscale Res Lett.

[8] Chu RL, Chiang TH, Hsueh WJ, Chen KH (2014). Passivation of GaSb using molecular beam epitaxy Y_2_O_3_ to achieve low interfacial trap density and high-performance self-aligned inversion-channel p-metal-oxide-semiconductor field-effect-transistors. Appl Phys Lett.

[9] Cheng S, Sang L, Liao M, Liu J, Imura M, Li H (2012). Integration of high-dielectric constant Ta_2_O_5_ oxides on diamond for power devices. Appl Phys Lett.

[10] Yan Z, Zhou C, Xiang Z, Peng Z, Dou Y, Wang W (2013). Passivation mechanism of thermal atomic layer-deposited Al_2_O_3_ films on silicon at different annealing temperatures. Nanoscale Res Lett.

[11] Zhang X, Tu H, Zhao H, Yang M, Wang X, Xiong Y (2011). Band structure and electronic characteristics of cubic La_2_O_3_ gate dielectrics epitaxially grown on InP substrates. Appl Phys Lett.

[12] Mcdonnell S, Pirkle A, Kim J, Colombo L, Wallace RM (2012). Trimethyl-aluminum and ozone interactions with graphite in atomic layer deposition of Al_2_O_3_. J Appl Phys.

[13] Suzuki M, Yamaguchi T, Fukushima N, Koyama M (2008). LaAlO_3_ gate dielectric with ultrathin equivalent oxide thickness and ultralow leakage current directly deposited on Si substrate. J Appl Phys.

[14] Zhang HT, Dedon LR, Martin LW, Engelherbert R (2015). Self-regulated growth of LaVO_3_ thin films by hybrid molecular beam epitaxy. Appl Phys Lett.

[15] Golalikhani M, Lei QY, Chen G, Spanier JE, Ghassemi H, Johnson CL (2013). Stoichiometry of LaAlO_3_ films grown on SrTiO_3_ by pulsed laser deposition. J Appl Phys.

[16] Yeluri R, Liu X, Guidry M, Koksaldi OS, Lal S, Kim J (2014). Dielectric stress tests and capacitance-voltage analysis to evaluate the effect of post deposition annealing on Al_2_O_3_ films deposited on GaN. Appl Phys Lett.

[17] Lim BS, Rahtu A, Gordon RG (2003). Atomic layer deposition of transition metals. Nat Mater.

[18] Batra N, Gope J, Vandana N, Panigrahi J, Singh R, Singh PK (2015). Influence of deposition temperature of thermal ALD deposited Al_2_O_3_ films on silicon surface passivation. AIP Adv.

[19] Park TJ, Sivasubramani P, Coss BE, Kim HC, Lee B, Wallace RM (2010). Effects of O_3_ and H_2_O oxidants on C and N-related impurities in atomic-layer-deposited La_2_O_3_ films observed by in situ x-ray photoelectron spectroscopy. Appl Phys Lett.

[20] Yao Y, Fu K, Yan C, Dai J, Chen Y, Wang Y, Zhang B, Hitz EM, Hu L (2016). Three-dimensional printable high-temperature and high-rate heaters. ACS Nano.

[21] Ma JW, Lee WJ, Cho M-H, Chung KB, An C-H, Kim H, Cho YJ, Moon DW, Cho HJ (2011). Changes in electronic structure of La_x_Al_y_O films as a function of postdeposition annealing temperature. J Electrochem Soc.

[22] Fan JB, Liu HX, Ma F, Zhuo QQ, Hao Y (2013). Influences of different oxidants on the characteristics of HfAlO_x_ films deposited by atomic layer deposition. Chinese Phys B.

[23] Cao D, Cheng X, Zheng L, Wang Z (2014). Effects of rapid thermal annealing on the properties of HfO_2_/La_2_O_3_ nanolaminate films deposited by plasma enhanced atomic layer deposition. J Vac Sci Technol A.

[24] Lee B, Park TJ, Hande A, Kim MJ, Wallace RM, Kim J (2009). Electrical properties of atomic-layer-deposited La_2_O_3_ films using a novel La formamidinate precursor and ozone. Microelectron Eng.

[25] Eom D, Hwang CS, Kim HJ, Cho MH, Chung KB (2008). Thermal annealing effects on the atomic layer deposited LaAlO_3_ thin films on Si substrate. Electrochem Solid-State Lett.

[26] Suzuki M (2012). Comprehensive study of lanthanum aluminate high-dielectric-constant gate oxides for advanced CMOS devices. Materials.

[27] Molina J, Tachi K, Kakushima K, Ahmet P, Tsutsui K, Sugii N (2007). Effects of N_2_-based annealing on the reliability characteristics of tungsten/La_2_O_3_/silicon capacitors. J Electrochem Soc.

[28] Maeng WJ, Kim WH, Kim H (2010). Flat band voltage (V_FB_) modulation by controlling compositional depth profile in La_2_O_3_/HfO_2_ nanolaminate gate oxide. J Appl Phys.

[29] Chiang CK, Wu CH, Liu CC, Lin JF, Yang CL, Wu JY (2011). Effects of La_2_O_3_ capping layers prepared by different ALD lanthanum precursors on flatband voltage tuning and EOT scaling in TiN/HfO_2_/SiO_2_/Si MOS structures. J Electrochem Soc.

